# A realist evaluation of the implementation of open visiting in an acute care setting for older people

**DOI:** 10.1186/s12913-019-4653-5

**Published:** 2019-11-21

**Authors:** Helen Hurst, Jane Griffiths, Carrie Hunt, Ellen Martinez

**Affiliations:** 1 Colloboration For Leadership in Applied Health Research Greater Manchester (NIHR), Greater Manchester, UK; 2 The University of Manchester, Manchester Academic Health Sciences Centre, Manchester University NHS Foundation Trust, Manchester, UK; 3 Elderly Health, Manchester Universter NHS Foundation Trust, Oxford Road, Manchester, M13 9WL UK

**Keywords:** Open visiting, Realist evaluation, Older people

## Abstract

**Background:**

Open visiting refers to the principle of unrestricted visiting hours in the hospital setting to enable relatives, families and carers to visit at any time. There has been recognition that open visiting supports the principle of patient and family supported care and improves communication. Despite this there has been difficulty in implementing open visiting and barriers identified. The aims of this study were therefore to evaluate the implementation of open visiting, the barriers to implementation, sustainability and the impact of open visiting on communication between health care professionals, families and carers.

**Methods:**

The study was conducted on two large acute wards for the older person. Realist evaluation methods were used to understand ‘what works well, how, for whom and to what extent.’ Mixed methods were employed including qualitative interviews and descriptive analyses of routine data sets**.** Following the methodology of realist evaluation, programme theories were identified a long with the context, mechanisms and outcomes of implementation, to better understand the implementation process.

**Results:**

The results of this study identified some key findings, demonstrating that open visiting does improve communication and can help to build trusting relationships between families/carers and health care professionals (HCP). Barriers to implementation were based on the belief that it would impinge on routines within the ward setting. To achieve the principles of patient and family/carer centred care, the key mechanisms are the confidence and skills of individual nurses and health care assistants to engage with relatives/carers, whilst retaining a sense of control, particularly when care is being delivered to other patients.

**Conclusion:**

In summary, open visiting creates a positive culture which fosters better relationships between families/carers and HCPs. Involving families/carers as partners in care does not happen automatically in an environment where open visiting is the policy, but requires engagement with staff to encourage and support relatives/carers.

## Background

Historically, in adult hospital settings visiting hours have been restricted to set times [1]. This was thought to be effective in protecting patients’ quiet times and to allow nurses and other health care professionals (HCPs) time to carry out duties and provide care [2]. Open visiting refers to the principle that visiting hours are not restricted in the hospital setting and relatives and carers can visit at any time. Despite unrestricted visiting being introduced in paediatric settings to improve the wellbeing and health of children [1], these values have not been transferred to adult settings.

There are some important principles when considering open visiting in adult hospital settings and each need to be considered. Firstly, the principles of patient and family centred care are at the heart of open visiting. Involving patients and families/carers in care planning and implementation is now supported as a safety initiative [3–5] and can also provide an environment that fosters equal partnership in health care delivery. Involving families and carers in care is particularly relevant for our aging population with increasingly complex health needs [6]. Reported deficits in their care is commonplace and health and care services have failed to keep up with this dramatic demographic shift; for hospitals the impact can be seen across all services [7]. A King’s Fund report highlighted the bed occupancy of this group: over 43% of non-elective admissions are over the age of 65 years, accounting for 53% of total bed occupancy. The report on in-patient care emphasises the importance of involving carers/family from admission to discharge [7].

Allowing access to visiting has been further emphasised by an initiative named ‘John’s Campaign’, which originated from poor care received by a patient with dementia. John’s campaign focusses on dementia care settings, enabling relatives and carers to have open access to visiting. Many NHS Trusts in the UK have signed up to the campaign and pledged to allow open visiting in this context. However, there are many other frail older patients who would benefit from having family members or carers present for longer periods than current visiting times allow [8].

The presence of family members or carers during the daytime is thought to help improve communication, especially in the care of the older person. Understanding the social and cognitive needs of patients involves a full comprehensive geriatric assessment (CGA) and ongoing discussion with families/carers. The CGA is a recommended standard for older people identified as frail admitted to hospital and has demonstrated improvements in outcomes and length of stay [9]. However, there has been increasing concern that restricted visiting does not promote the family/carer involvement in care which is recognised as important for patients’ recovery [2].

Secondly, the impact of open visiting and reported benefits to patients, families’/carers’ experience and improvements in outcomes, has not been studied in detail. Some reported benefits are increased satisfaction, improved communication and reduced anxiety of patients [2, 10, 11]. Reduction in complaints has also been reported, potentially as a result of improved communication [11].

The third principle relates to the attitudes of healthcare professionals, particularly nurses, which can impede the implementation and sustainability of open visiting [12]. These reported barriers relate to individual and workload effects, organisational and policy barriers (including lack of clarity), lack of education, support and training, and HCP desire for control over visiting hours [2, 12, 13]. In particular, there are reported concerns regarding interruptions, lack of privacy and lack of control over the environment underpinning a preference for set visiting times [13, 14]. Implementation therefore needs to be approached carefully and in consultation with the HCPs directly involved. For example, Derby NHS Trust in the UK [15] undertook a survey of 863 staff and visitors after the introduction of open visiting, identifying differences of opinion. Nurses in particular felt that there could be benefits such as quiet times, but restrictions were still considered necessary. The authors concluded that no real consultation had been conducted prior to the introduction; the lack of involvement regarding implementation led to problems in adoption.

Much of the published literature is from the USA which has a very different health care system to the UK, though similar themes were identified. The USA had a new statute introduced in 2011 to provide visitation privileges; this was a system-wide policy for a primary support person to have visitation access 24 h per day 7 days per week. A review by Nuss (2014) regarding the implementation of this statute identified that over 50% of hospitals did not have robust guidelines in place; however those that had introduced policies emphasised the importance of influencing cultural change [14].

Reports from the UK are promising and demonstrate individual hospital NHS Trusts implementing open visiting to enhance patient care but also to improve patient and family involvement in care. Unfortunately the studies conducted to date have involved small sample sizes and are not reported in detail [16].

Another important consideration is that the majority of research on open visiting has been conducted in intensive care units where traditionally patients are more acutely unwell, and therefore access for visitors more liberal. The methods previously used to evaluate open visiting were predominantly surveys and therefore key questions about why implementation of open visiting is still so difficult have not been addressed. There are also gaps in the literature regarding the impact of open visiting on outcomes such as falls. Whilst there is clearly a drive to consider open visiting, it is not universally accepted and barriers to implementation are an issue. The dichotomy is that nurses do see the benefits to open visiting but this is often outweighed by the fact that visitors are seen as a disruption [12].

The aims of this realist evaluation were to explore implementation of open visiting in an acute ward in order to understand what works, why it works and for whom it works.

Specific research questions included;

1. What are the barriers to implementing and sustaining open visiting in an acute ward?
2. How does open visiting impact on communication between patients, relatives/carers and staff in an acute ward?

## Methods

### Evaluation design and theoretical framework: realist evaluation

Open visiting can be viewed as a complex intervention due to the number of components involved. Realist evaluation is increasingly used in health research to evaluate complex interventions [17–20] and was first described by Pawson and Tilley [21]. It is theory-driven, based on the premise that to understand complex interventions in the ‘real world’ it is important to take into account social interactions and human behaviour [22].

A central component of realist evaluation is the development of programme theories, which are a set of statements describing how the programme is expected to cause its intended outcomes [23]. The focus is on building, testing and refining programme theories by exploring the complex dynamics of contexts (settings, organisational structures, programme participants), mechanisms (opportunities, resources and reasons or triggers which will make the programme work or not work) and outcomes, which may be intended or unintended depending on the link between the contexts and mechanisms [24, 25]. The aim is to present configurations of the contexts, mechanisms and outcomes (C-M-O) which reflect the results of the evaluation and the refined programme theories. Realist evaluation utilises appropriate methods of data collection to provide a clear process to test the programme theories. Outcomes are often not predefined and iteration may be necessary if outcomes are identified as the evaluation progresses [26]. The theoretical framework for this study is based in realism, however as the aims were also focused on implementation, other theories were considered. The Normalisation Process Theory (NPT) provides a framework to make sense of difficulties in implementation and change. It identifies factors which either promote or inhibit the incorporation of complex interventions into routine practice [27]. There are four main components to NPT: coherence (or sense-making), cognitive participation (or engagement), collective action (work done to enable the intervention to happen) and reflexive monitoring (formal and informal appraisal of the benefits and costs of the intervention) [27]. Sense-making and engagement were critical components in this study, and the planning of the implementation of open visiting was a crucial element of the process.

The three broad phases, therefore, for realist evaluation followed during this study were developing, testing and refining the programme theories; in this instance the programme is open visiting [23]. These processes will be described along with the data collection methods used within the study.

This study was guided by the reporting standards of RAMSEES II [28]. RAMSEES II is a guiding protocol for researchers to follow to ensure the realist evaluation methods and application adhere to quality guidelines.

#### Setting

The study took place in a large NHS Trust in the North of England. The Trust has three hospital sites. One of the smaller sites in the Trust had already introduced open visiting as a pilot on one ward. Although no formal evaluation was conducted at the time, open visiting continued because of the positive impact recognised by staff, patients, relatives and carers.

Two large acute medical wards for older people were chosen for the implementation and evaluation of open visiting. These wards were chosen because of the average age of inpatients and extended length of stay, representing people in hospital who might benefit from open visiting. The majority of patients were > 65 years old with a high prevalence of dementia; the average age of inpatients was 84 years. The average length of stay was 92 days. The ratio of nursing staff to health care assistants (HCA) was 1:1.5; on average 14 registered nurses compared to 21 HCA’s. Each ward had 28 beds; one ward cared for female patients and the other male.

#### Participants

Study participants included all stakeholders from the ward areas involved in the implementation of open visiting: medical, nursing, AHPs (allied health professionals), patients, relatives and carers.

#### Ethics

Full ethical approval was obtained through the Health Research Authority and approval gained from the Trust Research Department.

#### Phase one: development of programme theories and implementation of open visiting

In the initial stages of the project a steering group was convened and document created in order to provide clear, timely goals and a shared vision for implementation. The group included representation from the wards, a university lecturer, dementia specialist nurse, representative from the research and innovation division, the clinical effectiveness lead for the service and a matron involved in the pilot project. The team reflected broad experience with appropriate expertise for the project. The group met twice before implementation and once following data collection.

The initial stages involved meetings with stakeholders (12 in total) to bring together a process of engagement and awareness of the project. These initial meetings enabled development of the programme theories, a set of statements detailing how the programme was expected to cause its intended outcomes [23]. Raising awareness was part of the pre-implementation plan; this included provision of information leaflets and development of a patient and visitors’ charter to be displayed. A short presentation was devised and presented to staff before project implementation. This occurred during a 6-week period and the information sheets and charter were given to staff to read. The ward managers displayed the new open visiting charter and times at the entrances of the ward; these were in place at the time of implementation. Questionnaires were developed based on previous pilot work, the literature review and stake holder comments. These were distributed to staff 6 weeks before implementation to establish current views and beliefs about open visiting. Questionnaires were distributed via a link sent to individual email addresses, co-ordinated by the Trust Patient Experience Team. There were also paper copies within the ward areas. The questions can be viewed in Additional file 1.

The programme theories, described in Table 1, were developed through the initial stakeholder meetings and review of the literature. Within each statement the context, mechanism and outcome have been identified.

**Table 1:** Initial Programme Theories

Programme Theories	
1. Open visiting facilitates flexible access to health care professionals (HCP’s) (C + M) including medical, nursing and therapists’. This helps to build trust and improve communication (O).	
2. If relatives/carers are present more at busy times (C) it may become burdensome for staff because of a lack of control (M) over activities and interruptions. This is a potential barrier to successful implementation of open visiting (O).	
3. If relatives are present more with open visiting (C) they can become partners in care and be involved more in planning, implementing and delivering aspects of care (M). It may potentially impact positively on reducing harm and improving quality care (O).	

*C* Context, *M* Mechanism, *O* Outcome

#### Phase two: testing the programme theories

Once the programme theories had been identified the process of data collection was planned. Mixed methods, using both qualitative and quantitative approaches were used as described below.

### Qualitative interviews

Interviews were conducted with HCPs, patients and relatives/carers. Two sets of semi-structured interview guides were used for both groups (Additional file 2). Interviews were conducted by two researchers, audio-recorded, and transcribed verbatim. Neither of the researchers were part of the teams involved or working in the areas where the evaluation took place.

Semi-structured interviews were utilised to gain in-depth understanding of the mechanisms and context of the intervention**,** and to allow the flexibility to explore new themes or ideas as they arose. Topics were related to the programme theories, context, mechanism and outcomes.

A total of 30 interviews were conducted, with details of participants displayed in Table 2. The sampling strategy was purposive to include a wide range of HCP’ s within the multidisciplinary team. Patients’ relatives and carers were approached, given an information leaflet and asked if they wished to participate. Interviews were completed when the sample was sufficiently varied and data saturation was reached.

**Table 2:** Participants

Participant	Key in presented interview data	Numbers and (percentage)
Health Care Professionals		
Medical	M	3 (18%)
Nursing	N	8 (50%)
HCA (health care assistants)	HCA	3 (18%)
AHP	AHP	2 (12%)
Total		16
Relatives	Rel	9 (64%)
Patients	P	5 (36%)
Total of all Interviews		30

### Quantitative data (routine)

Descriptive data is routinely collected across the organisation in relation to quality, patient experience and safety outcomes. For the purpose of this study no additional data was collected. The data analysed was therefore obtained from incident reports of patient safety measures by predefined categories. These were pressure ulcers, falls, documentation, communication, safeguarding, diet/nutrition, behaviour/abuse and medication errors, as well as complaints and compliments. The team gathered numbers of each of these incidents for quantitative analysis. Whilst each incident report includes a narrative account completed by the reporting staff member, these were not included in the analysis. However, narratives attached to reported complaints and compliments were analysed qualitatively. The complaints and compliments were listed under broad pre-set headings: communication, treatment, discharge and medication.

The clinical effectiveness team provided data 6 months prior and 6 months post implementation. The decision to examine this data was based on stakeholder interest as to whether open visiting would have a direct impact on these outcomes.

#### Data analysis

**Fig 1:**
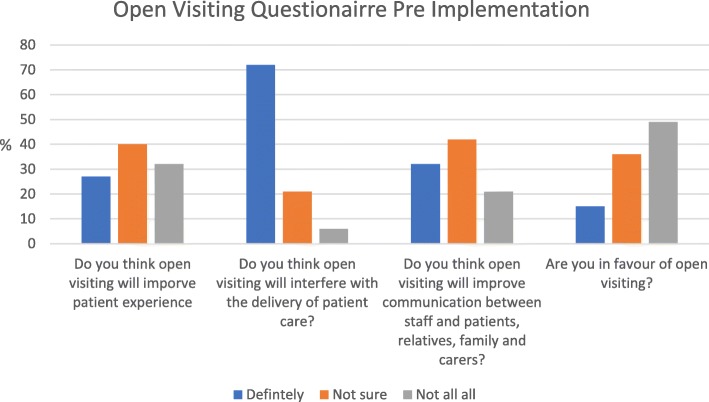
Pre Implementation Questionairre

Descriptive analysis of pre-implementation questionnaires is presented in Fig. 1. For each question an answer of ‘definitely,’ ‘not sure’ or ‘not at all’ was selected by the participant.

The qualitative data was coded following a process of familiarisation and emerging patterns to develop categories. Codes were then mapped to categories and sub-categories [29]. The next stage of the process was to scrutinise the sub-categories using the theoretical framework and initial programme theories. The categories were then fed into a matrix that included the headings of context, mechanisms and outcomes. So, for example a quotation (from a sub-category) which related directly to communication or trust was listed under that heading alongside the context, mechanism and outcomes. A group of four researchers took part in an afternoon workshop to configure and critique the contexts, mechanism and outcome (C-M-O) configurations and further refine the programme theories. Multiple outcomes were identified, often more than one in relation to each context and mechanism. The refining of the programme theories was then undertaken to identify the three basic C-M-O configurations presented in Table 3.

**Table 3:** Final Programme Theories

Context	Mechanism	Outcome
Health care professionals communicate more with families and carers Medical staff are receptive and available on ward rounds	Families/carers share information opportunities to discuss more of shared communication. The key mechanism related to more opportunities to communicate but the trigger was the openness of the HCP	Reduced complaints Less meetings needed to be arranged with families/carers Building of trust and shared decision making
HCPs do not feel confident about asking families/carers to leave whilst care is being delivered	The key mechanism and trigger for this negative outcome was the lack of confidence and lack of use/knowledge of available resources e.g. leaflets and charters.	Privacy and dignity is not maintained Staff feel pressured in the presence of families/carers.
HCP’s are receptive and include relatives/carers in aspects of care	The mechanism related to the openness and willingness of HCP and confidence to engage with relatives/carers encouraging participation	Improved nutrition Improved taking of medication Reduced anxiety of patients Improved quality of care

## Results

The routine data collected by the clinical incident team regarding the numbers of incidents relating to each category did not significantly increase or decrease pre and post implementation, with one exception. This was in relation to falls, which were reduced from 50 in the pre-implementation period to 27 post-implementation on one ward. This will be discussed in detail within the combined results section to further understand the context and mechanisms relating to this reduction. The number of complaints recorded during the time period of the study were minimal: on one ward there were eight complaints recorded pre-implementation and nine post-implementation, on the other ward 11 were recorded pre-implementation and nine post-implementation.

Table 2 details the participants interviewed, including a variety of healthcare professionals. Only small numbers of patients were able to participate in the interviews which was a reflection of their age and acute illnesses.

The results of the questionnaire distributed pre-implementation are presented in Fig. 1. A total of 47 questionnaires were completed, with data presented as a percentage response to each answer. The questionnaire results provide some insight into the barriers to open visiting. The most significant response can be seen from the question regarding open visiting interfering with routine care with 70% of responses being ‘definitely.’ 49% of respondents were not in favour of open visiting pre-implementation, with only 15% in favour.

The findings will now be presented including verbatim quotations from interviews. Broad thematic headings have been used, linking to the theoretical framework. The aim is to give an overall description of what works for whom, how and why.

### Communication and trust

Improved levels of communication were often described by HCPs. This was related to increased interactions as relatives and carers were more present during the day, which was of particular relevance to the allied health professionals (AHPs) who deliver therapy sessions in the mornings. The following excerpts demonstrate this:

*Okay, my thought about it is very positive, yeah, because I’ve noticed some changes when it has been introduced. For example – I’m talking about nurse point of view – we are more close to relatives. We have more chance to talk with them. We have more chance to listen their concerns. We have more time to spend with them to talk about the patient altogether, nurses, relatives and … I mean families and patient. Yeah, I’m very impressed (N 002).*

*I think it’s really good. Yeah, I think families are enjoying it. And it’s good for us, as a speech therapist, to be able to sort of grab family generally if they’re on the ward more often. So we get to see people and get a better baseline for our patients and it’s been useful (AHP 004).*

Staff commented on the continuity of the interactions, and being able to provide up-to-date information to families and carers.

*Initially as doctors we were sceptical thinking we would be plagued with interruptions and requests to see relatives all the time but overall that hasn't been the case in fact communication has improved (M 011).*

One of the hypotheses for this study was that complaints would reduce, as the majority of complaints are related to communication. Although the number of complaints reported officially through the routine systems did not appear dramatically different, the interviews highlighted many benefits in this regard. Complaints are managed on a daily basis by staff and often resolved without recourse to official routes; staff felt that the increased presence of relatives/carers, with more frequent opportunities to discuss care, was hugely beneficial in this area.

### Barriers to open visiting

From the early stakeholder meetings and questionnaires, it was clear that all levels of staff perceived barriers to open visiting, in particular concerns about interruptions to daily activities within the ward environment. Such beliefs held by staff are perhaps the influencing factors determining whether open visiting will be successful and yet following implementation these perceptions and beliefs were often changed. From the interviews, it was clear that this was an important contextual issue, so the HCPs’ receptiveness to open visiting and engagement with relatives/carers would influence both the implementation process and the outcomes. Pre-implementation, medical staff were particularly concerned that too many interruptions would hold up their ward rounds. However, this did not prove to be the case, with some medical staff changing their views or even challenging their own beliefs post-implementation when they realised the benefits of seeing relatives more frequently meant less meetings were required. As described here:

*It's a brilliant idea because being a part of a medical team it is so easy to get the information round to families and next of kin. You don’t have to wait for a particular time or arrange a meeting, they just come in, you update them, being proactive and patients’ relatives are so happy (M 006).*

Some staff felt that open visiting did impinge on privacy and dignity for toileting and meals. Eating in front of other relatives/carers was found to be embarrassing for some patients, an issue to be taken into consideration. Similarly with toileting, not all patients can access the toilet and instead use a commode at their bedside. Maintaining dignity for these patients is very important as these quotations represent:

*And mealtimes, you know, not everybody's happy to eat their meals when there's other people, especially people that they don't know, visiting other people, they're not happy eating their meals in front of strangers (Rel 007).*

*Yeah, and I think if people … For instance, if a patient wanted the toilet, they would feel embarrassed to go to the toilet even on the commode or on a bed pan when there is visitors … .. So you’re talking about sort of dignity in care ? Yeah, I mean we have the right to ask them to leave, but the amount of time these ladies need the toilet it’s just … it’s a bit … it’s not practical really, you know (HCA 003).*

However, there were examples of how this could be overcome on the pilot ward where open visiting was originally introduced, where staff designed small signs to hang in areas stating that care was being delivered (a message also covered in the open visiting charter and leaflet). Furthermore, some staff commented that mealtimes with relatives’ present had in fact proven beneficial. These examples of differences in staff views only emphasise further the individual influences staff can have. It is also relevant to the numbers of clinical support workers who deliver the majority of personal care to patients. If they do not feel confident or are not empowered they may find the presence of relatives burdensome.

### Family and carer involvement in care

The involvement of families and carers in care is an organisational and National driver to improve standards, in particular for older people with dementia [6, 7]. This study demonstrated that this is not straightforward and does depend hugely on the confidence and skills of individual nurses and HCPs. This quotation demonstrates that in particular, family or carer assistance with hygiene needs may cause staff to feel uncomfortable, whereas assistance with medication or meals is viewed differently:

*Hygiene I have only seen one or two family members be involved not many are involved because they don’t want to but, compliance with medication has increased I would say that (N 004).*

Interestingly, it may be lack of confidence in asking for help, as this quotation from a relative illustrates:

*Well, I've come now and she's going to have a wash, and they've asked me to come to the dayroom. They've not asked me to help. I did get her pyjamas out what I thought she might want to put on. And a few times I've come and, you know, she's got …*

*well, she's not got her glasses on today and she can't see a thing without them. And her hearing aids weren't in the other day, and she can't hear a thing without them. So I've done those things when I've come, you know, put them on and found her hearing aids and things (Rel 006).*

Although leaflets and charters were produced to provide information and explanation around these issues they were not fully used by staff to share with relatives/carers. Not using these to their full advantage may have influenced how some of the staff dealt with different situations. Staff however felt that open visiting was appropriate for patients at the end of their life or patients with dementia. Context can be an important influencing factor, for example the patient’s condition and their requirements. Balancing some of the pressures experienced by staff is clearly described here:

*For example, sometimes dedicate a proper time to family members takes time for other things. So the nurse should be in the position to say, sorry, I need to prioritise my work – at the moment I cannot spend time with you because I’ve got another urgent work to do. And sometimes it can happen that they do not understand that we’ve got priorities (N 007).*

The relative / carer perspective showed awareness of the competing pressures on staff members as this quotation explains:

*We try to be, you know, as unobtrusive as possible. We know that the nurses and the doctors have things to do and we will try and stay in the background. But there again, if there is anything that we can observe and help, and make them aware of, I feel that's integral to what we would do (Rel 001).*

One of the aims of this study was to understand the impact of open visiting on reducing harm such as falls. Interestingly, the number of falls reported through the standard reporting systems varied greatly, and one ward did see a reduction post-implementation. However, it is difficult to determine what influenced the reduction of falls. One change in the context of the ward where falls were reduced was a new ward manager who approached supervision of patients at risk of falling differently and introduced alternative ways of working. It became clear during the interviews that other important patient outcomes such as nutrition and medication concordance improved with the introduction of open visiting. These factors are not measured routinely by the organisation and would be a difficult outcome to measure. Patient and family/carer feedback from this evaluation was wholly positive; the flexibility of open visiting allowed relatives and carers to work around their own schedules. They commented on various aspects of open visiting; below are examples, including views on helping at mealtimes:

*I think it's a really good idea, particularly, you know, if you've got a large family, it means that you can have them staggered out during the day, than everyone trying to come for an hour and a half, you get very tired or, you know. I think that way they don't feel as separated from the family (Rel 009).*

*… .definitely think with the nutrition. I know the experience when my mum had a stroke a few years ago, they had protected mealtimes. And I actually said to them, if I could come in and sit with her, I could get her to eat far more than you just putting a tray in front of her. I understand you've got people that need more help than she did, she could feed herself but she needed encouragement. And I think that would make a big difference, yeah (Rel 009).*

Protected meal times were introduced into clinical areas in the Trust to attempt to provide a focus for staff and were targeted at reducing meal-time interruptions by members of clinical staff. The presence of family members during meal times might improve patients’ nutritional intake as explained by the excerpt above.

#### Revised programme theories

Following the analysis, the revised programme theories were organised into C-M-O configurations (see Table 3). This was not a linear process but rather a theoretical construct; the refined programme theories have been included in the table to demonstrate this, noting also the changes from the original theories.

## Discussion

The aim of this study was to undertake a realist evaluation of the implementation of open visiting on an acute ward for older people. Open visiting as a policy allows family members and carers to have an increased presence at the patient’s bedside. A major focus of healthcare is to provide patient and family centred care. Policy and reports in the UK nursing literature regarding the implementation of open visiting emphasise the key message of ‘improving patient and family centred care and enabling family/carers to be more involved in care’ [11]. Such reports are often press releases with minimal outcome data reported. However, despite evidence that presence of families/carers can reduce anxiety and improve patient outcomes, implementation of open visiting has proven difficult [14, 30]. Attitudes and beliefs of nursing staff have been demonstrated to influence implementation, with many concerns expressed in relation to control over access and timings [14, 16, 31, 32]. The current study highlighted such concerns from the initial questionnaires and interviews, in particular in regards to interference with routine care. This may be due to an underlying fear of family members being present, with healthcare providers needing to demonstrate accountability in the presence of families and carers.

Confidence was an issue raised in this study; when staff lacked the confidence to ask relatives/carers to leave at times of personal care they perceived a compromise of dignity to patients. The availability of a charter and information for relatives/carers detailing when they might be asked to leave could support staff in this situation, although during this study it became clear that these were not always used. This highlighted that the availability of information materials does not in itself aid staff to develop the necessary skills to deal with such situations.

The involvement of relatives/carers in active care is often described as positive, with benefits to both patients and staff [10, 32]. Family and carer involvement in care needs does not happen automatically and requires trusting and confident staff to engage. Therefore, a key contextual issue for successful implementation is education and support of staff. The mechanisms required to achieve this include not only provision of leaflets and charters but enabling staff to develop their skills in communicating with relatives/carers. This will enable the HCP to build trusting therapeutic relationships which educate, involve and comfort families and carers. This study demonstrates some of the complexities surrounding family/carer involvement, however this is only the beginning of the cultural shift necessary to provide the right environment and support to enable this. This view is supported by others regarding the care of older people in particular [7] and the Francis Report emphasised this in detail [33]; it is therefore imperative we continue to examine ways in which we can improve and embrace patient and family/carer experience and involvement in care. An important improvement demonstrated by the implementation of open visiting was increased levels of communication with HCPs. Although a focus of this study was reducing complaints, this is not the only benefit. Staff in this study reported more interactions and opportunities to share information. Such increased communication between the nurses and medical team has been shown to have positive outcomes as it contributes to further insights into patients’ wishes [31, 34]. In many hospital settings HCAs deliver the majority of personal care to patients so it is therefore important to include them in any implementation, ensuring that they feel confident in dealing with relatives and carers.

Many of the challenges that have been discussed in relation to implementation of open visiting have to be considered. Future work to enable this to become standard practice must take into account all of these factors. Prior to implementation of open visiting the key components of the NPT described earlier were considered. Coherence (or sense-making) and cognitive participation (or engagement) was the focus of presentations and development of written material. The collective action (work done to enable the intervention to happen) was also a focus before implementation and was supported by managers once implemented. The final component of reflexive monitoring (formal and informal appraisal of the benefits and costs of the intervention) was collected as part of the interviews. Cost analysis was not applied to this study but is an important outcome to be considered. The cost benefit is likely to be observed over a longer period of time when outcomes relating to length of stay, for example, can be directly linked to open visiting. Despite the engagement processes the HCPs made decisions as they experienced the presence of family and carers whist delivering care.

A further hypothesis-generated theory for this study was that open visiting would improve outcomes relating to patient harm. Although there was a reduction in falls on one ward, this was likely related to the presence of a new ward manager who implemented changes to managing patients at risk of falling. This involved alteration to how staff worked within the environment, with use of more observable positions throughout the day. Health outcomes relating to open visiting are poorly reported; most studies are qualitative and report improvements in satisfaction and experience. Interview data from this study identifies potential improvements in nutrition when relatives/carers are present at meal times, and reference to medication-taking was made though limitations in the data collected cannot substantiate true cause and effect. A recent communication from NHS England highlights improved outcomes where open visiting has been introduced, including within mental health services, emphasising improved outcomes relating to falls, nutrition, communication and improved transitions of care. Key measures of success are collaboration with carers and senior leadership in implementation [35]. Future research in this area should consider measures to improve health care professionals’ confidence and skills in order to allow families/carers and patients to plan and deliver care together, in particular HCA’s who are involved in all aspects of personal care.

In answering the key questions for this study based on the realist evaluation theory of what works, for whom, in what circumstances and why, there were some clear take home messages. Open visiting worked for patients and their relatives because it offered flexibility and enabled many relatives and carers to be present for longer periods. For the HCPs, open visiting was successful if they were engaged and confident in dealing with relatives and carers; the key mechanisms were down to individuals who felt confident.

Future work and the introduction of open visiting across NHS Trusts therefore needs to take into account these findings and recommendations including recent communication from NHS England. Trust-level and senior leadership need to drive the changes and support staff in all areas. Ward managers are pivotal in bringing about the changes and must be engaged from the beginning. As evidence builds and more communication of the benefits are published, providing them with these messages will be vital. Tool kits, information and training will all enable changes to culture and promote family/carer involvement.

### Strengths and limitations

One of the strengths of this study was the use of realist evaluation as a way to understand what works for whom and in what circumstances. This enabled a deeper understanding of the contexts and mechanisms that influence the outcome following the implementation of open visiting. There are limitations to this study, as firstly only two wards were used for implementation, specifically for acute care of the older patient. The age of the patients, their acute illness and high levels of dementia influenced patient participation in this study and only small numbers were recruited**.** Details of participants’ levels of independence were not collected and may have provided useful information for better understanding the context. The post-implementation study period was only 6 months which may have influenced the results. A further evaluation in 12 months’ time would be beneficial to refine and further develop the programme theories. Although this study was conducted in acute medical wards, one of the strengths has been understanding how future implementation strategies could be shaped to scale up and introduce open visiting across other clinical areas. However, among the stakeholders and staff interviewed there was a consensus that open visiting is possible in all ward areas, and some NHS Trusts have adopted this policy. The pilot ward has continued to have open visiting for over 2 years and it has since been implemented across the hospital site.

## Conclusion

Open visiting was clearly received positively overall, and although at this early stage improvement to outcomes such as falls has been difficult to demonstrate, other improvements including communication, reduction of complaints and improvement of patient, relative and carer experience were observed. Open visiting fosters an environment which nurtures trust and confidence and encourages family and carer participation. This evaluation was relatively short; routine data on patient safety outcomes along with patient and relative reported outcomes (through experience data routinely collected) will continue to be examined. Roll-out across the organisation will require a strategy to ensure staff are engaged, using recommendations from other sources (including video testimonies of the benefits) will be crucial. Ensuring signage, leaflets and a charter are visible for all will also be an essential component of implementation.

## Supplementary information

**Additional file 1.** Questionnaire pre-implementation.

**Additional file 2.** Interview Guide for Staff.

## Data Availability

The datasets used and/or analysed during the current study are available from the corresponding author on reasonable request.

## Abbreviations

AHP: Allied health professional
C-M-O: Context- Mechanism- Outcome
HCA: Health care assistant
HCP’S: Health care professionals
NHS: National Health Service
NPT: Normalisation Process Theory
RAMSEES: Realist And Meta-narrative Evidence Syntheses: Evolving Standard
UK: United Kingdom
USA: United States of America
