# Supplementary material for: A realist evaluation of the implementation of open visiting in an acute care setting for older people
Source: BMC Health Serv Res. 2019 Nov 21;19:867. doi: 10.1186/s12913-019-4653-5 (PMC6873458; doi:10.1186/s12913-019-4653-5)
Supplement: Supplementary file 1 — Additional file 1. Questionnaire pre-implementation. [file 12913_2019_4653_MOESM1_ESM.docx]

Additional file 1

Questionnaire pre-implementation

Three options were given to tick: Definitely, Not Sure, Not At All

1. Designated role
2. Do you think open visiting will improve the patient experience?
3. Do you think open visiting will interfere with the delivery of patient care?
4. Do you think open visiting will improve communication between staff and patients, relatives, family and carers?
5. Are you in favour of open visiting?

Comments open box included
