# Supplementary material for: A realist evaluation of the implementation of open visiting in an acute care setting for older people
Source: BMC Health Serv Res. 2019 Nov 21;19:867. doi: 10.1186/s12913-019-4653-5 (PMC6873458; doi:10.1186/s12913-019-4653-5)
Supplement: Supplementary file 2 — Additional file 2. Interview Guide for Staff. [file 12913_2019_4653_MOESM2_ESM.docx]

Additional file 2

Interview Guide for Staff

Can you tell me your thoughts on open visiting?

Can you describe how it has impacted on you as a person?

Can you tell me what you think the impact is for patients, relatives, carers?

Can you discuss why and how you think it has worked (or not)?

Can you discuss what you would change about the process of implementation?

Do you think this is something that could be replicated, if so what do you think should be considered?

Interview Guide Patients, relatives, carers

Can you tell me what you think about open visiting?

What do you think the advantages/disadvantages are to it?

What difference to you think it has made to you as a (patient), (relative/carer)?

Do you think it should be open visiting in all wards?

Can you talk about any differences you have noticed in particular communication with staff?
